# Learning from safety incidents in high-reliability organizations: a systematic review of learning tools that could be adapted and used in healthcare

**DOI:** 10.1093/intqhc/mzab046

**Published:** 2021-03-17

**Authors:** Naresh Serou, Lauren M Sahota, Andy K Husband, Simon P Forrest, Robert D Slight, Sarah P Slight

**Affiliations:** School of Pharmacy, Newcastle University, King George VI Building, Newcastle Upon Tyne, Tyne and Wear NE1 7RU, UK; Operating Theatres, Singleton Hospital, Swansea Bay University Health Board, Swansea SA2 8QA, Wales, UK; Swansea Medical School, Swansea University, Swansea SA2 8QA , Wales, UK; School of Pharmacy, Newcastle University, King George VI Building, Newcastle Upon Tyne, Tyne and Wear NE1 7RU, UK; School of Pharmacy, Newcastle University, King George VI Building, Newcastle Upon Tyne, Tyne and Wear NE1 7RU, UK; Department of Sociology, Durham University, Durham DH1 1SZ, UK; Population Health Sciences Institute, Newcastle University, Baddiley-Clark Building, Richardson Road, Newcastle upon Tyne, Tyne and Wear NE1 7RU, UK; Department of Pharmacy, Newcastle upon Tyne Hospitals NHS Foundation Trust, Freeman Rd, High Heaton, Newcastle upon Tyne, Tyne and Wear NE7 7DN, UK; School of Pharmacy, Newcastle University, King George VI Building, Newcastle Upon Tyne, Tyne and Wear NE1 7RU, UK; Population Health Sciences Institute, Newcastle University, Baddiley-Clark Building, Richardson Road, Newcastle upon Tyne, Tyne and Wear NE1 7RU, UK; Department of Pharmacy, Newcastle upon Tyne Hospitals NHS Foundation Trust, Freeman Rd, High Heaton, Newcastle upon Tyne, Tyne and Wear NE7 7DN, UK

**Keywords:** safety incidents, simulation, debriefing, after action review, crew resource management, high-reliability organizations

## Abstract

**Objective:**

A high-reliability organization (HRO) is an organization that has sustained almost error-free performance, despite operating in hazardous conditions where the consequences of errors could be catastrophic. A number of tools and initiatives have been used within HROs to learn from safety incidents, some of which have the potential to be adapted and used in healthcare. We conducted a systematic review to identify any learning tools deemed to be effective that could be adapted and used by multidisciplinary teams in healthcare following a patient safety incident.

**Methods:**

This review followed the Preferred Reporting Items for Systematic Reviews and MetaAnalyses for Protocols reporting guidelines and was registered with the PROSPERO (CRD42017071528). A search of databases was carried out in January 2021, from the date of their commencement. We conducted a search on electronic databases such as Web of Science, Science Direct, MEDLINE in Process Jan 1950-present, EMBASE Jan 1974-present, CINAHL 1982-present, PsycINFO 1967-present, Scopus and Google Scholar. We also searched the grey literature including reports from government agencies, relevant doctoral dissertations and conference proceedings. A customized data extraction form was used to capture pertinent information from included studies and Critical Appraisal Skills Programme tool to appraise on their quality.

**Results:**

A total of 5921 articles were identified, with 964 duplicate articles removed and 4932 excluded at the title (4055), abstract (510) and full-text (367) stages. Twenty-five articles were included in the review. Learning tools identified included debriefing, simulation, crew resource management and reporting systems to disseminate safety messages. Debriefing involved deconstructing incidents using reflective questions, whilst simulation training involved asking staff to relive the event again by performing the task(s) in a role-play scenario. Crew resource management is a set of training procedures that focus on communication, leadership and decision-making. Sophisticated incident-reporting systems provide valuable information on hazards and were widely recommended as a way of disseminating key safety messages following safety incidents. These learning tools were found to have a positive impact on learning if conducted soon after the incident with efficient facilitation.

**Conclusion:**

Healthcare organizations should find ways to adapt to the learning tools or initiatives used in HROs following safety incidents. It is challenging to recommend any specific one as all learning tools have shown considerable promise. However, the way these tools or initiatives are implemented is critical, and so further work is needed to explore how to successfully embed them into healthcare organizations so that everyone at every level of the organization embraces them.

## Introduction

One of the principles of human performance is that people are fallible, and even the most experienced and well-trained people make mistakes [1, 2]. This principle applies to every organization, with individual behaviour influenced by processes and values. It is also acknowledged that error-likely situations are predicable, manageable and preventable, by understanding the reasons why mistakes occur and applying the lessons learned [3, 4]. A safety incident is defined as any unplanned, undesired event that hinders the completion of a task and may cause injury, illness or property damage, or a combination of all the three in varying degrees from minor to catastrophic [5]. In high-reliability organizations (HROs), such as aviation, nuclear power operations and chemical industries, team learning is a widely established cultural practice used to reflect upon and review processes following safety incidents [5, 6]. HROs are known to function nearly error-free in extremely challenging and uncertain environments [1, 5] and have a number of key characteristics, including (i) individuals with expertise are given decision-making responsibilities during emergencies irrespective of their hierarchical position within the organization. (ii) management by exception, where managers only get involved with operational decisions as and when required, (iii) climate of continuous training, (iv) use of several channels to communicate safety critical information, (v) in-built redundancy, including the provision of back-up systems in case of a failure and (vi) frequent engagement with frontline staff to gain broader view and entire perspective of operations [7]. They attain high safety standards by applying principles such as pre-occupation with failure, which includes giving attention to minor or small indicators which may cause potential problems and use incidents and near misses as pointers to measure their system’s or organizations strength and condition [7]. Although this preoccupation with failure is a key principle of HRO, it is often ignored in healthcare [8]. Retrospective reviews and incident reports have highlighted how healthcare staff often assumes that what is in front of them is correct and do not approach tasks with a mindset to look for potential errors [9–12].

Similar to traditional HROs, healthcare organizations experience frequent patient safety incidents, and, in recent years, the number of avoidable patient safety incidents in healthcare continues to grow [1]. According to a recent report from NHS Improvement England, 21 898 186 patient safety incidents occurred between October 2018 and September 2019 [13]. These incidents varied from no patient harm to significant patient harm or patient death. Up to 10% of patients experience preventable adverse events in hospitals worldwide [3]. These estimates demonstrate that hospitals are ‘high-risk systems’ [1, 3, 7] and, in more recent years, initiatives have been undertaken to transform hospitals into HROs as these organizations have been characterized by low probability of errors and adverse events [1, 5, 14]. A culture of continuous learning and open communication is some of the HRO principles that have been adapted and implemented successfully in healthcare organizations [5, 15–17]. However, healthcare organizations have got socio-organizational barriers, with clinical decision-making often shared between health professionals and their patients and the need to adapt to individual patients’ needs [1, 5, 7, 14].

Healthcare organizations are known to experience challenges when attempting to reduce their number of safety incidents [1]. These include a lack of understanding among healthcare staff about what incidents should be reported, how they will be analysed and fear that punitive action may be taken against anyone regarded as culpable [18]. Due to the nature of clinical work patterns and pressures, it has also been reported that healthcare workers and management do not have sufficient time for team learning following serious incidents [19, 20]. A recent review of the impact of surgical incidents on healthcare staff working in operating theatres emphasized the need to deconstruct serious incidents in surgical environments so as to understand the reason(s) why they occurred and apply the lessons learnt [21]. It highlighted the need for a cultural change to team learning and an emphasis on team-based approaches to help hospital staff intervene earlier to prevent these incidents from re-occurring.

A number of learning tools and initiatives have been used within HROs to learn from safety incidents; they tend to fall into two broad categories of approach involving either simulation and/or debriefing [20, 22–27]. Debriefings are usually facilitated by experienced psychologists and trained senior staff soon after the incidents [15]. They are very structured sessions, with every member of the team given an equal opportunity to discuss the event, and the learning from the sessions shared with the rest of the staff [27]. In aviation, simulation is used as a tool for aircrew to learn from incidents [27]. Simulation and debriefing have been used in healthcare mostly in education setting but not widely used to learn from patient safety incidents [16, 27, 28]. The aviation industry have also built and used data systems to track progress of safety incidents, such as feedback and safety messages following safety incidents [4]. A previous systematic review, which identified the key characteristics of HROs discussed above, did not specifically explore the learning tools or initiatives that were used in the various HROs following safety incidents [7]. More learnings should be taken from HROs, and so this systematic review explores what tools or initiatives have been used in HROs and whether they can be adapted for use in the healthcare sector to learn from safety incidents.

## Methods

This review followed the Preferred Reporting Items for Systematic Reviews and MetaAnalyses for Protocols (PRISMA-P) reporting guidelines and is registered with the PROSPERO database (CRD42017071528). We defined a practical tool as a learning process or a method used to learn from safety incidents. We included all articles that met the following inclusion criteria:

- Primary research articles or reviews that describe a practical tool or initiative to help deconstruct safety incidents for learning purposes.
- Any learning tool used in any HROs, high hazard industries or safety critical industries.
- Studies using any type of research method.
- Any unpublished articles, conference proceedings, editorial comments.
- Any articles that did not describe a tool or initiative in detail and focused more on learning theories or were not available in English were excluded.

### Search strategy and study selection

**Figure 1:**
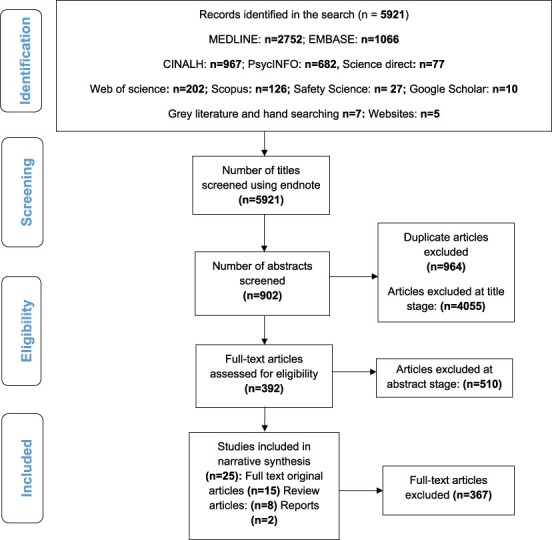
PRISMA diagram: representation of the steps involved in the search strategy.

We developed a comprehensive and broad set of search terms, which included both MeSH terms and text words, with the input of the university librarian. We carried out a simple search using key concepts, such as ‘high reliability’, ‘high reliability organizations’, ‘high dependable organizations’, ‘high standard organizations’, ‘high dependable organizations’, ‘high standard organizations’, ‘high reliability companies’, ‘high reliability industries’, and ‘high reliability bodies’ in different databases to find relevant articles and see how they were indexed using controlled vocabulary. We repeated these for a number of different articles to see what subject headings have been used. A list of MeSH terms and Boolean operators used in the electronic databases is provided as a supplementary material 1. The following electronic databases were searched in January 2021, from the date of their commencement: Web of Science, Science Direct, MEDLINE in Process (Ovid) Jan 1950-present, EMBASE (Ovid) Jan 1974-present, The Cumulative Index to Nursing and Allied Health Literature (CINAHL) 1982-present, PsycINFO 1967-present, Scopus and Google Scholar. We also searched the grey literature including reports from HRO websites, such as www.high-reliability.org; www.hse.gov.uk; https://psnet.ahrq.gov; https://safetymatters.co.in/; https://llis.nasa.gov/and government agencies such as National Patient Safety Agency (NPSA), and Local and Regional Clinical Commission Groups. Any relevant doctoral dissertations and conference proceedings identified in the grey literature (http://www.opengrey.eu), and reports from NPSA, Association for Perioperative Practice, Institute for Health Improvement, Local and Regional Clinical Commission Groups were reviewed. The Institute for Health improvement and other particular groups have had an interest in the successful strategies used in other industries to help evaluate, calculate and improve the overall reliability of complex systems. Further material was sought by scanning reference lists in the included articles. Searches were also carried out within specific academic journals (e.g. safety science and organization science) in order to identify any relevant papers in press or recently available. Duplicate articles were removed using Endnote reference management tool version X7.7.1. Studies identified as potentially relevant for inclusion were assessed independently by two reviewers (N.S. and L.M.S.), with arbitration by a third reviewer (S.P.S.), if necessary. This involved reviewing the titles, abstracts and full texts, and documenting the reason why each article was excluded. Figure 1 represents the PRISMA diagram illustrating the steps involved in the search strategy.

### Data extraction and synthesis

A customized data extraction form, provided as a supplementary material, was developed and included the authors’ names, year of study, country where the research was conducted, research methods used, tool or initiative described, what the purpose of the tool was, what types of population the tool was used for, how the tool facilitated learning in terms of mechanism by which it worked and how well it worked, and a risk of bias (quality) assessment of each article. A narrative synthesis of the data was undertaken by two authors (N.S. and L.M.S.) [29]. First, a preliminary synthesis was undertaken to develop an initial description of the results of included studies. Then, the reviewers moving beyond identifying and tabulating results to further explore relationships within and across the included studies, such as how and why a particular learning tool worked in different circumstances in various HROs. Finally, overarching themes and subthemes relating to the research aims were identified independently by two reviewers (N.S. and L.M.S.) [30]. Third author (S.P.S.) was used to check for consistency and approval of the final themes emerged from the studies. The quantitative data and reports from the included articles were summarized and analysed for recurrent patterns across other qualitative studies and articles. Table 1 gives details of the initial subthemes and overarching themes extracted.

**Table 1:** Subthemes and themes extracted from each included article, reviews and reports

Articles	Initial subthemes/tools discussed	Overarching themes/learning tools
Allen *et al*. [34]	After action review, debriefing, incident review, post-incident review	Debriefing
Crowe *et al*. [40]	After action review, debriefing, incident review	Debriefing
Eddy *et al*. [33]	Debriefing, post-brief, after incident review	Debriefing
Ellis and Davidi [46]	After action review, after incident review, post-brief	Debriefing
Ford *et al*. [45]	Crew resource management, non-technical skills	Crew resource management
Garvin [54]	Debriefing, after action review, post-incident review	Debriefing
Lardner and Robertson [47]	Simulation, simulation scenarios, technology	Simulation
Marquardt *et al*. [42]	Crew resource management, non-technical skills, crisis skills	Crew resource management
Mastaglio *et al*. [36]	Debriefing tool: after action review, post-brief, review after incident	Debriefing
Mavin, Kikkawa and Billett [44]	Simulation, technology, simulators	Simulation
Nergard [41]	Debriefing, post-flight debriefing, incident debriefing	Debriefing
Roth [43]	Debriefing, after incident review, post-brief	Debriefing
Salter and Klein [37]	Debriefing, after action review, incident review	Debriefing
Scott et al. [35]	Debriefing, after action review, post-brief, incident review	Debriefing
Taylor and Robertson [39]	Crew resource management, non-technical skills, crisis skills, safety skills, cockpit skills	Crew resource management
Review articles and reports		
Allen [49]	Debriefing, incident review	Debriefing
Jeffrey, Mitchell and Everly [51]	Debriefing, critical incident stress debriefing, brief after trauma, supporting skills	Debriefing
Kaps *et al*. [53]	Crew resource management, non-technical skills, crisis skills	Crew resource management
Helmreich [48]	Simulation, line-oriented flight training, technology, real event activities, learning skills	Simulation
Helmreich [16]	Simulation, technology	Simulation
Megan *et al*. [56]	The safety reporting, learning system, advanced incident reporting system, disseminating safety messages	Reporting and dissemination of safety messages
Oudheusden *et al*. [26]	Technology for reporting and technology for learning and disseminating messages	Reporting and dissemination of safety messages
Rolfsen [50]	Operational debrief, crew debrief, post-incident review	Debriefing
Schindler *et al*. [54]	Debriefing, post-project review	Debriefing
Tannenbaum and Cerasoli [55]	Debriefing, incident review, post-activity review, learning	Debriefing

### Risk of bias (quality) assessment

A Critical Appraisal Skills Programme (CASP) tool for qualitative, quantitative and systematic reviews was used to access the quality of qualitative, quantitative papers and systematic reviews, respectively. This CASP tool consists of 10 questions that each focus on a different methodological aspect of the study. Two reviewers (N.S. and L.M.S.) carried out quality appraisal of each article independently. Any disagreements were resolved by discussion with a third additional reviewer (S.P.S.), if needed. The scores and quality of the selected quantitative and qualitative papers were included in the data extraction table provided as a supplementary material 2. CASP scores were used to distinguish studies of relative higher and lower qualities. The qualitative studies were also assessed for the use of methodological triangulation (use of two or more methods), which has been advocated as a way of safeguarding the ‘validity’ of qualitative studies [31, 32].

## Results

A total of 5921 articles were identified, with 964 duplicate articles removed and 4932 excluded at the title (4055), abstract (510) and full text (367) stages. Twenty-five articles were included in the final review (15 primary research articles, eight review articles and two reports). Figure 1 provides a diagrammatic representation of the search strategy used. The 15 primary research studies were conducted in six countries: USA (*n* = 8) [33–40], Europe (*n* = 2) [41, 42], Australia (*n* = 2) [43, 44], New Zealand (*n* = 1) [45], Israel (*n* = 1) [46] and United Kingdom (*n* = 1) [47]. Of these 15 articles, eight used quantitative method [33–35, 39, 42, 45–47], six qualitative methods [36–38, 41, 43, 44] and one-mixed methods [40]. Two of the six qualitative selected articles used more than one of the following methods of data collection, including observations, formal and informal interviews, recorded debriefing sessions, observed and recorded simulator sessions. The four remaining qualitative studies used only one method.

The eight quantitative studies [33–35, 39, 42, 45–47] had heterogeneity of the data and interventions used, with different outcomes, study designs (e.g. surveys and questionnaires), populations, interventions (e.g. AAR model or simulation) and settings (military, fire department, aviation), and therefore, it was not possible to conduct a meta-analysis. Table 1 represents the themes and sub-themes that were extracted from selected papers. We identified four overarching themes from the included articles: simulation, debriefing, crew review management (CRM) and dissemination of safety incidents. Within each overarching theme, simulation, CRM and reporting systems to disseminate safety messages were identified as individual tools and they are discussed accordingly as sub-themes. We also found various HROs used debriefing as an approach and were identified as separate tools such as after action review (AAR), post-flight debrief, Mitchell model and post-project review, which we discuss further below under the debriefing theme.

### Simulation

Simulation has been used in HROs to deconstruct and learn from safety incidents [16, 44, 47, 48]. The term ‘simulation’ refers to a model of a real activity created for training purposes. A typical simulation model consists of seven sequential steps: introduction, simulation briefing, theory input, scenario briefing, scenario, debriefing and ending [16, 43, 47]. The scenarios are usually based on real life or past events and are typically facilitated by experienced facilitators [47]. Four studies explored the impact of simulation in learning after safety incidents in HROs. Engineers from British petroleum industry and aviation crew at all levels from different aviation sectors were included in these studies.

In the aviation sector, it is mandatory for flight crews to take part in simulation following a significant safety event, such as the incident in which a passenger flight in Canada crashed after only few seconds because it was not able to reach adequate altitude beyond the end of the runway, due to ice and snow on the wings [16]. The subsequent simulation exercise was found to be effective in changing flight crews attitudes and behaviour and helping them recognize the importance of human performance limiters (such as fatigue and stress) and adequate aircraft maintenance [16]. In the maritime sector, British Petroleum (BP) used simulation after a safety event in the Gulf of Mexico as a result of an oil leak [47]. These mandatory simulation events enable the crew to relive the event again by performing the tasks in a role-play and sharing the subsequent learning and recommendations [47]. Some of the participants described how this approach was a ‘useful way to gauge thoughts and decisions’ and a ‘better way to discuss [an] incident’ [47]. Along with technical aspects, simulation was found to be beneficial in training staff on non-technical skills such as teamwork, communication, prioritization, leadership and situation awareness [16, 47].

### Debriefing

The term ‘debriefing’ refers to conversational sessions that involve seeking the views and understandings of individuals after a specific event [41, 43, 47, 49]. Debriefing sessions have been widely used by soldiers at all levels in the military, pilots and air crew in aviation, fire fighters in fire departments, engineers and workers in railways and chemical industries and are normally carried out soon after the event. We also found various HROs used debriefing either on its own or as part of simulation-based learning to help deconstruct and learn from safety incidents. Different debriefing tools were identified such as post-flight debrief, Mitchell Model post-project review and AAR, the latter using four main questions: ‘What was supposed to happen? What actually happened? Why were there differences? What can you learn from this experience?’ These sessions were facilitated by observers/controllers who used probing questions to elicit responses, such as ‘talk me through it’ and ‘how did that work?’, or photographs with probes such as ‘What do you see? What’s going on here?’ [37]. Fire Fighters at the eastern USA, who were often offered AAR after any fire rescue operation, were surveyed on their experience, with one participant explaining how it allowed them ‘to say something without retribution.’ [40].

In the aviation industry, team-based ‘debriefing’ sessions took place both before and after flight take off where an experienced senior member of staff and a trained psychologist provided feedback on the technical and non-technical performance of the flight members, respectively [16, 41, 44, 49, 50, 52, 54]. One participant described how: ‘Normally the operative debriefing is straightforward and amounts to declaring that everything went according to normal operations. Occasionally, we need time to work through specific events that occurred during the flight, either in the cockpit or in the cabin. The debriefing will then continue to its conclusion with no regard to time’ [41]. The Mitchell model has been used to enhance resistance to stress reactions or help individuals ‘bounce back’ from a traumatic experience [51]. It includes seven elements: introduction, fact, thought, reaction, symptom, teaching and re-entry [51] and is slightly different to AAR debriefing as the personal experiences of the affected individual, including the impact of and their reaction to the incident, are discussed in detail.

### Crew resource management

HROs such as aviation, military and automotive industries also developed crew resource management (CRM) training programmes, which were complementary to the simulation-based team training with debriefing sessions, but put more emphasis on non-technical skills [53]. These included effective leadership, teamwork, dealing with diverse personalities and operating styles, workload management and situational awareness; preparation, planning, and vigilance; workload distribution, distraction avoidance; individual factors, and stress reduction [53]. A US study showed significant improvement in safety, efficiency, dependability and assertiveness amongst aviation managers following CRM training [39]. Similarly, a New Zealand study found significant improvements on flight attendants’ and cabin crews’ understanding of each other’s role and responsibilities, their roles in flight emergencies, and their perception of safety, following CRM training. These improvements were evaluated and measured using the Flight Safety Attitudes Questionnaire in the study [45]. The study also found that joint training sessions, where flight attendants and pilots work together to find solutions to in-flight emergency scenarios, provided a particularly useful strategy in breaking down communication barriers [45]. A German study also found a significant improvement in teamwork-related attitudes and workers’ situational awareness after the CRM training program [42].

### Reporting and dissemination of safety messages

The reporting and dissemination of safety messages to staff is also viewed as an effective learning process following an event [26, 34, 38, 46, 49, 55, 56]. Incident reporting systems provide valuable information on hazards and the potential risk that these hazards may actually cause harm; this is useful for organizations as they can learn from previous incidents and implement interventions to reduce these risks. HROs such as nuclear and radiation power plants developed sophisticated incident-reporting systems to record and improve organizational learning from incidents [26, 56]. For example, the radiotherapy institute in USA developed the Safety Reporting and Learning System for Radiotherapy, which allowed users to submit their own incident reports to the system, as well as search and review reported incidents about similar technologies, procedures or near misses so as to learn from others who have experienced them [56]. Similarly, the Belgian Nuclear Research Centre used a sophisticated incident reporting system named Retour d’Experiences to share reported incidents and safety messages to staff within their nuclear centre [26], thus promoting collective learning and safety governance. Staff expressed their satisfaction in using the system as key learning points and active causes of the incidents were often analysed [26].

## Discussion

### Statement of principal findings

The findings of this study are timely, given the recent report published by the World Health Organization (WHO) on the Patient Safety Incident Reporting and Learning systems, which highlighted the significance of using and developing learning systems following patient safety incidents [57]. This review shows that debriefing, simulation, CRM and systems to disseminate safety messages following safety incidents were positive tools and approaches for learning. Simulation has been used in HROs to train staff on technical and non-technical skills and debriefing used to help deconstruct and learn from safety incidents. CRM put more emphasis on non-technical staff skills, while sophisticated incident reporting systems helped record and improve organizational learning from incidents. The effectiveness of learning and staff satisfaction in using debriefing and simulation appeared to depend on the facilitator and the environment in which the sessions were organized and conducted. The contents and structure of the learning tool was as important as the facilitation of these sessions. They also needed to be conducted in a safe environment for staff to discuss and reflect on the incident and encourage efficient team teaching and learning.

### Strengths and limitations

To our knowledge, this the first systematic review to explore the tools and approaches used in HROs to learn from safety incidents and give recommendations as to how these approaches could be used in healthcare context. We identified learning tools used in a wide variety of HROs such as aviation, military, fire department, automobile industries, chemical, petroleum, nuclear and radiation industries. We excluded a number of studies that focused solely on learning theories, as they did not concentrated on learning tools or initiatives per se, but rather the wider cultural barriers that exist in bringing about change. Although outside the scope of this review, these studies may have provided further insights and recommendations for future learning. We acknowledge that the inclusion of some more targeted library databases (e.g. the Association for Computing Machinery database or the American Society for Testing and Materials Standards and Engineering Digital Library) might have been useful. While it would have been impossible to search all relevant library databases, some important research may have been missed.

### Interpretation within the context of the wider literature

Healthcare has used simulation as an educational tool for training staff on clinical interventions, such as acute management of patients in emergency and in basic and advanced life support programmes rather than for the sole purpose of deconstructing and learning from safety incidents [22, 58–60]. Previous studies have highlighted how staff working patterns, staff shortages and time pressures made simulation training a challenging prospect in healthcare [60–63]. We recommend that organizations take account of these important barriers and explore how to better adapt and embed these tools into healthcare organizations. The different casual factors, which contribute to a real incident, could be replayed in simulation for the staff to reflect and learn as a team without compromising patient safety.

Few studies have been conducted to explore the usage of AAR structured format for debriefing sessions in the healthcare context. The WHO implemented five steps of safer surgery, including a briefing and ‘debriefing’ before and after the surgery [64], the benefits of which have been well reported [65–67]. However, there have been inconsistencies between ‘what’ the surgical community viewed as an ‘effective’ debriefing and actual practice [66, 67]. Ahmed *et al.* found that debriefing was often conducted in an unstructured way following surgery and feedback focused more on the negative than positive aspects of individuals’ performance [66]. Competitive professional culture, clinical and educational commitments and lack of time were found to be the main barriers for conducting debriefings after surgery [66–68]. More work needs to be done in training health professionals on debriefing techniques and more effective facilitation.

This review identifies the importance of non-technical skills and their contribution towards learning from safety incidents. These include the social, cognitive and personal management skills that enable safe and effective work performance, by enhancing the individual’s communication, situation awareness and decision-making and managing stress and fatigue while working in HROs. Several studies in the healthcare sector have identified poor non-technical skills as significant contributing factors for patient safety incidents especially in operating theatres [59, 69]. Similar to how simulation has been used in healthcare as an educational tool, some clinical specialities have also devised training programmes based on CRM components [59, 69–72]. Using CRM to help deconstruct and learn from safety incidents in healthcare could lead to a greater understanding of the importance of non-technical skills and improvements in safety [69, 71, 73]. CRM was adapted to healthcare, resulting in care improvement and harm reduction across a wide variety of medical specialties [53, 74–76]. When implemented in the operating rooms, CRM has been shown not only to improve communication and staff morale but also to reduce patient morbidity and mortality [53, 74, 76, 77]. However, unlike HROs, these programmes do not appear to be mandatory in the healthcare sector [72, 76–79], and more work is required to adapt these learning approaches in staff development training and undergraduate and postgraduate medical and surgical education curriculums. Furthermore, healthcare organizations could support the utilization of learning tools in a number of ways, including ensuring that teams have the necessary time to engage in this activity and involving trained facilitators and psychologists in the debriefing process, the importance of which has been highlighted in previous studies [27, 80].

### Implications for policy practice and research

Various studies in safety science and quality and safety stressed that one or two initiatives or learning tools alone are not sufficient to address safety incidents nor is there a ‘one-size-fits-all’ solution [1, 5, 6, 19, 20, 24, 81–86]. It is challenging to recommend any specific interventions as all learning tools have shown considerable promise for positive learning. Healthcare organizations should be encouraged to use a combination of methods to help staff learn from safety incidents. NHS improvement recognized the various steps involved in a patient safety incident [10], including the reporting of the incident, further investigations conducted into why it happened and certain changes put in place to prevent the incident occurring in the future [10]. The learning tools identified in this review could be used at the different stages of this incident process. For example, simulation can be used to help staff understand how they need to act in real-life situations and allows them to fine-tune both their technical and non-technical skills in a safe environment. Similarly, sharing safety messages following incidents in daily team meetings will increase staff awareness and help them become more vigilant. Although used effectively in HROs, learning tools, such as debriefing and simulation, have been used inconsistently in healthcare, with several disparities reported around conducting debriefing sessions following surgery [27, 66, 67, 87]. Staff workload, staff shortages and lack of time and resources were all viewed as major barriers in using these tools [23, 66, 88]. HROs have prioritized safety over other goals, allocating extra staff and resources where needed and relaying a consistent message that safety is as or more important than other business objectives [1]. Healthcare organizations need to endorse these HRO principles by prioritizing continuous learning and safety at work place. HROs also promote ‘mindful leadership’ and identify any gaps between how managers think that procedures should be used and how they are actually applied by frontline staff [8]. Similar to HROs, healthcare leaders need to identify these gaps and promote a culture of learning within their organization in order for any tools, discussed in this review, to be effective.

## Conclusions

HROs have adopted a variety of learning approaches following safety incidents, and studies stress that using one or two learning tools alone are not sufficient to address safety incidents. Healthcare organizations should be encouraged to use a combination of methods to help staff learn from safety incidents. Healthcare organizations should adapt the learning tools used in HROs following safety incidents; however, the way these tools or initiatives are implemented is critical. Further work is need to explore how to successfully embed them into healthcare organizations so that everyone at every level of the organization embraces them. Leaders within healthcare need to promote a culture of continuous learning and psychological safety for these learning tools to be effective.

## Supplementary Material

mzab046_Supp

## Data Availability

No new data were generated or analysed in support of this review.
