# Supplementary material for: Learning from safety incidents in high-reliability organizations: a systematic review of learning tools that could be adapted and used in healthcare
Source: Int J Qual Health Care. 2021 Mar 17;33(1):mzab046. doi: 10.1093/intqhc/mzab046 (PMC8271183; doi:10.1093/intqhc/mzab046)
Supplement: mzab046_Supp [file mzab046_supp.zip › Supplementary material Data extraction sheet revised final.doc]

| **Serial no:**  **Supplementary material 2: Data extraction sheet** | **Author & Year** | **Country** | **Research methods** | **Practical tool** | **Purpose and the population the tool is used** | **Results and outcomes**  **(How tools are used for learning)** | **Risk of bias (quality) assessment of each article using CASP** |
| --- | --- | --- | --- | --- | --- | --- | --- |
| **Research Articles** | | | | | | | |
| 1) | Allen, J. A. et al (2010) | USA | Quantitative | Debriefing : After Action Review (AAR) | This study investigated the role of after-action reviews on perceptions of safety climate at the group  and organizational levels by promoting team learning  **Population:** Fire Fighters | After-action reviews create a definite setting through which managers can promote safety climate in high-risk environments. | 8/10 |
| 2) | Crowe J et al (2017) | USA | Mixed Methods:  Quantitative study and Qualitative | After Action Review (AAR) –Debriefing tool | To investigate what makes for good  and bad after-action reviews (AARs)  **Population:** Firefighters | AARs provides a venue for team building and potentially enhancing the safety climate on crews. | 8/10 |
| 3) | Eddy, E. R et al. (2013) | USA | Quantitative | Debriefing | Aim of the research is to compare two types of team-led debriefs:  1) Unguided version  2) Guided version that integrates lessons learned from research and practice.  The study investigated how team-led debriefs  influence team processes and, in turn, team effectiveness and individual-level  outcomes  **Population:** Business students for strategic management course | “Guided” team-led debriefs can yield greater benefits than unguided debriefs.  The study shown moderate  boosting team processes and performance. | 8/10 |
| 4) | Ellis, S., & Davidi, I. (2005) | Israel | Quantitative | Debriefing : After action review | Using Quasi-field experiment the study aim to examine the importance of AAR in debriefing both failures and success following an event for team learning and performance.  **Population**: Military, soldiers | Performance of soldiers doing consecutive navigation exercises improved considerably when they were debriefed through AAR on their failures and successes after each training day, compared with others who reviewed their failed events only | 8/10 |
| 5) | Ford. J et al (2014) | New Zealand | Quantitative | Crew resource management | The study aim to explore the effects of Crew Resource Management (CRM) training on flight  attendants' safety attitudes and team learning  **Population:** Aviation- Flight attendants | CRM training for flight attendants is a valuable learning tool for increasing positive teamwork behaviours between the flight attendant and pilot sub-groups.  - Joint training sessions, where flight attendants and pilots work together to find solutions to in-flight emergency scenarios, provide a particularly useful strategy in breaking down communication barriers | 8/10 |
| 6) | Garvin D.A (2000) | USA | Qualitative review | Debriefing: After Action Review | To explore the facilitation of AAR in Military  **Population:** Military | The study has given number of recommendations in how to facilitate AAR which included broad participation, structured process, skilled facilitation , need for attention to recordings of th event and dissemination.  -The study also recommended a climate of openness and candour along with consistency while facilitating AAR’s | 8/10 |
| 7) | Lardner R & Robertson I (2011) | UK | Quantitative | Simulation: Simulation Scenarios | The study aim to investigate whether individual learning occurred, leading to changes in  individual knowledge, routines and performance following simulation exercises  - Whether team and organisational learning occurred, leading  to changes in team and organisational knowledge, routines  and performance following simulation exercises .  **Population**: Engineers and managers from British Pertoleum | The feasibility of constructing a  simulated incident scenario, with the same generic features as real incident.  -The scenario was successfully used to determine  the extent to which individual and team/organisational  learning had occurred via post-incident briefings.  Further the study found that using the more active scenario method, coupled with group discussion  and individual and group feedback on performance, led to new knowledge and a changed approach towards situations with similar features. | 6/10 |
| 8) | Marquardt, N et al (2010) | Germany | Quantitative | Crew resource management | The aim of the study is to develop, implement, and evaluate crew resource management (CRM) training program specifically designed for employees  within the automotive industry  **Population**: Staff within automotive industry | The results showed a significant improvement in a wide range of CRM-relevant categories, especially in teamwork-related attitudes, in addition to an increase in the workers’ situational awareness after the training program. | 8/10 |
| 9) | Mastaglio .T et al. (2011) | USA | Qualitative | Debriefing tool: After Action Review | The study aim to explore the current practice and theoretical foundations  of the After Action Review  **Population**: Military | The key findings from this research show that AAR is a fundamental part of the Army training culture with recognized value at the trainer and trainee level. A reference model, the Integrated Theory of AAR (ITAAR) is described together with educational and information dissemination recommendations that will strengthen Army-wide expertise. . | 7/10 |
| 10) | Mavin J. T, Kikkawa Y & Billett S (2018) | Australia | Qualitative | Simulation | -The study aims to explore the effect of simulation followed by debriefing and analysed over 32 biannual simulator training and  assessment sessions for learning.  -The study further explored the quality and outcomes of  the learning experiences, particularly the efficacy of the simulator-to-debriefing ratio.  **Population:** Aviation, flight crew | -Simulation sessions are useful to practice the real event.  -Factors such as personal readiness of the pilots and situational factors during the training need to be considered while facilitating the debriefing sessions following simulation exercises. | 8/10 |
| 11) | Nergard V (2015) | Norway | Qualitative: Case study | Debriefing : post-flight debriefing | The purpose of this study was to explore how pilots potentially learn from their and others’ experience  through post-flight debriefing  **Population:**  Aviation ;flight crew | The post-operative debriefing, helped the crew  to create an emotional distance to the event and allowed the participants to reflect and learn from the event. | 8/10 |
| 12) | Roth W.M (2015) | Australia | Qualitative  Observations, formal and informal interviews, recorded debriefing sessions, observed and  recorded simulator sessions | Debriefing tool | -To investigate the cultures and cognition of debriefing in the aviation industry to learn from events.  -Explored how the debriefing meeting was organised in terms of duration and participation following the debrief and simulation sessions.  **Population:** Aviation; Pilots | -There was substantial difference on the amount of talk that the flight examiners and crewmembers contributed to the debriefing meetings.  -Study showed that pilots tend to be aware that they forget much of the detail and it therefore influenced the debriefing session and their participation.  -Less experienced pilots found it more useful to have the flight examiner narrate back to them what they had done and tell them why it had been wrong and how to improve upon it. themselves.  -The study showed that the cultural practice of debriefing depends on its structure, on the cognitive  organization of the flight examiner notes and memory. | 8/10 |
| 13) | Salter M. S  Klein. G.E (2007) | USA | Qualitative | Debriefing: After Action Review | The purpose of this research was to examine the conduct of After Action Reviews (AARs) at the Combat Training  Centers (CTCs).  **Population:** Military | Even the best-trained  Observer/Controllers (O/Cs) tend to err on the side of providing too much information while facilitating AAR  -The study provided with recommendations which  Included a prototype AAR rating scale that could be used as a job aid, performance checklist, or as an instructional tool during O/C training. | 7/10 |
| 14) | Scott C (2013)et.al. | USA | Quantitative | Debriefing :After Action Review | Study examined the impact of post-incident, prediscussion  ambiguity and freedom of dissent on participant satisfaction with AARs  **Population:** Fire Fighters | The level of post-incident, pre-discussion  ambiguity was negatively related to AAR satisfaction. --Freedom of dissent, however, attenuated the  negative influence of ambiguity on AAR satisfaction. | 8/10 |
| 15) | Taylor .J.C &Robertson M.M (1995) | USA | Quantitative | Crew resource management | The study explored the effects of crew resource management (CRM) training in airline maintenance  **Population:** Aviation, Flight crew | Following CRM training, there is significant improvement in manager’s attitude towards safety, efficiency, dependability and assertiveness. | 7/10 |
| **Review articles and reports** | | | | | | | |
| 16) | Allen, J. A(2018) | USA | Critical review article | Debriefing | Review looked at how debriefing is used in various organisations to promote learning and performance across the various services.  Review also investigated the factors relevant to debrief effectiveness and the outcomes for individuals,  teams, and organizations that deploy debriefs.  **Population:** the fire service, aviation, education, and in a variety of organizational training and simulation environments. | The article provided with recommendations in how best to implement debriefs from a practical perspectives in various high reliability organisations. | NA |
| 17) | Jeffrey T. Mitchell J.T and Everly G.S (1993) | USA | Report | Debriefing tool: Critical incident stress debriefing (CISD) | Critical Incident Stress Debriefing (CISD) is a seven phase, small group supportive crisis process. It is just one of the many crisis intervention techniques which are included under the umbrella of a Critical Incident Stress Management (CISM) program.  **Population:** Law enforcement, fire service, emergency medical, military, aviation and other specialized professions | A Critical Incident Stress Debriefing can best be described as a psycho-educational group process.  It was reported to be an efficient process to learn following the event. It involves structured group story-telling process with applied information to group member reactions to a critical incident and facilitate their recovery. | NA |
| 18) | Kaps et al (1999) | USA | Literature Review | Crew resource management | -The purpose of this study was to review and synthesize the  literature and research on CRM training  -To capture and report these major changes in recent CRM programmes.  **Population:** Aviation, Military | -Key topics of the review included  a) the current status of CRM training and research, b) evolution of CRM concepts,  c) measuring methods, and d) application of CRM. | 7/10 |
| 19) | Helmreich RL(1999) | USA | Review | Simulation: Line –oriented flight training (LOFT) | To review the High Fidelity simulator in CRM training. The review explored the effect of high fidelity simulators in CRM training | -High-fidelity simulators consist of a cockpit with working instruments  and controls, the sensation of motion and a visual representation of the environment outside the cockpit windows.  -CRM has expanded the use of the simulator as a training tool. It enabled crews to test themselves in tackling complex problems—ranging from bad weather to mechanical failures—that cannot be resolved by simply following a procedure outlined in the flight manual. | NA |
| 20) | Helmreich RL (2000) | USA | Report | Simulation | To explore the how lessons are learned in aviation  **Population:** Aviation | In aviation, accidents are usually highly visible, and as a result aviation has developed standardised methods of investigating, documenting, and  disseminating errors and their lessons | NA |
| 21) | Megan TD et al ( 2017) | USA | Feature Article | The Safety Reporting and Learning System for Radiotherapy (SAFRON)  -Advanced Incident reporting system | The Safety Reporting and Learning System for Radiotherapy (SAFRON)aims to increase safety in radiation therapy facilities by learning from reported events and decreasing the likelihood of a similar event occurring.  - Use near misses and incidents to promote a culture of safety in  the radiation therapy  department  **Population**: Staff within Radiation therapy department | Allowed users to submit their own incident reports to the system, as well as search and review reported incidents and near misses at other facilities. | NA |
| 22) | Oudheusden et al (2017) | Belgium | Review article | Technology for reporting and learning : Retour d’Experiences (REX) | Documentary analysis of incident  reports, interviews, and focus groups with personnel, it illustrates how REX  enacts a safety governmentality centered on identifying incident causes and for learning  **Population**: Staff within Nuclear plant | Retour d’Experiences (REX) to share reported incidents and safety messages to staff within their Nuclear centre, thus promoting collective learning and safety governance. Staff expressed their satisfaction in using the system as key learning points and active causes of the incidents are often analysed. | NA |
| 23) | Rolfsen J (2013) | Norway | Review article | **Operational (Crew) Debrief:**  The model is named “APPROACH”  **A** -Debrief **ASAP**, if necessary on the aircraft and away from distractions  **P**- **Participation** with all crew involved  **P**- **Purpose**,. The purpose is to provide accurate, factual and objective operational information after an incident to ensure mutual understanding  **R** **Review** the known particulars about the incident  **O** Explain the **operational** significance of the incident, including any safety implications  **A** **Ask** if there are any questions from the crew  **C** **Check** for understanding  **H Help** crew to access company medical or psychological services if required. | **Purpose:** Tool for preventing the accumulation of bad experiences and the development of over-determined reactions following an adverse event in aircraft and for team learning.  **Population:**  Aviation ;flight crew | The tool is used to give the air company a wealth of information on incidents  - The tool gave the crew an opportunity to discuss about the event and gave qualified personnel present in the debrief the chance of identifying if the crewmembers need any additional support  -It gave each crewmember valuable information about the safety related  aspects of the occurrence | Not applicable (NA) |
| 24) | Schindler M et al (2003) | Switzerland | Review article | Debriefing tool: Post Project review | This article presents an overview of proven methods to record experiences from projects and discusses their use in project management. | The article discusses central project debriefing problems such as the lacking willingness to learn from mistakes.  -Gives recommendations on how debriefing processes can be integrated successfully into project procedures. | NA |
| 25) | Tannenbaum, S. I., & Cerasoli, C. P. (2013) | USA | Systematic review and meta-analysis | Debriefing | The review aim to assess the efficacy of debriefs with a quantitative review  **Population**: Aviation, Military | -The review found that organizations can improve individual and team performance by approximately 20% to 25% by using properly conducted debriefs.  -The review further identified that debriefs are a relatively inexpensive and quick intervention for enhancing performance. | 9/10 |
