# Supplementary material for: Learning from safety incidents in high-reliability organizations: a systematic review of learning tools that could be adapted and used in healthcare
Source: Int J Qual Health Care. 2021 Mar 17;33(1):mzab046. doi: 10.1093/intqhc/mzab046 (PMC8271183; doi:10.1093/intqhc/mzab046)
Supplement: mzab046_Supp [file mzab046_supp.zip › Supplementary material document search terms260121.doc]

**Supplementary material 1:** List of MeSH terms and Boolean operators used in the electronic databases

| 1 | learning.mp. [mp=ti, ab, ot, nm, hw, fx, kf, ox, px, rx, ui, an, sy, tn, dm, mf, dv, kw, dq, tc, id, tm, mh] |
| --- | --- |
| 2 | education.mp. [mp=ti, ab, ot, nm, hw, fx, kf, ox, px, rx, ui, an, sy, tn, dm, mf, dv, kw, dq, tc, id, tm, mh] |
| 3 | teaching.mp. [mp=ti, ab, ot, nm, hw, fx, kf, ox, px, rx, ui, an, sy, tn, dm, mf, dv, kw, dq, tc, id, tm, mh] |
| 4 | tutoring.mp. [mp=ti, ab, ot, nm, hw, fx, kf, ox, px, rx, ui, an, sy, tn, dm, mf, dv, kw, dq, tc, id, tm, mh] |
| 5 | organi*ational learning.mp. [mp=ti, ab, ot, nm, hw, fx, kf, ox, px, rx, ui, an, sy, tn, dm, mf, dv, kw, dq, tc, id, tm, mh] |
| 6 | workplace learning.mp. [mp=ti, ab, ot, nm, hw, fx, kf, ox, px, rx, ui, an, sy, tn, dm, mf, dv, kw, dq, tc, id, tm, mh] |
| 7 | individual learning.mp. [mp=ti, ab, ot, nm, hw, fx, kf, ox, px, rx, ui, an, sy, tn, dm, mf, dv, kw, dq, tc, id, tm, mh] |
| 8 | team learning.mp. [mp=ti, ab, ot, nm, hw, fx, kf, ox, px, rx, ui, an, sy, tn, dm, mf, dv, kw, dq, tc, id, tm, mh] |
| 9 | 1 or 2 or 3 or 4 or 5 or 6 or 7 or 8 |
| 10 | framework*.mp. [mp=ti, ab, ot, nm, hw, fx, kf, ox, px, rx, ui, an, sy, tn, dm, mf, dv, kw, dq, tc, id, tm, mh] |
| 11 | intervention*.mp. [mp=ti, ab, ot, nm, hw, fx, kf, ox, px, rx, ui, an, sy, tn, dm, mf, dv, kw, dq, tc, id, tm, mh] |
| 12 | model*.mp. [mp=ti, ab, ot, nm, hw, fx, kf, ox, px, rx, ui, an, sy, tn, dm, mf, dv, kw, dq, tc, id, tm, mh] |
| 13 | tool*.mp. [mp=ti, ab, ot, nm, hw, fx, kf, ox, px, rx, ui, an, sy, tn, dm, mf, dv, kw, dq, tc, id, tm, mh] |
| 14 | design*.mp. [mp=ti, ab, ot, nm, hw, fx, kf, ox, px, rx, ui, an, sy, tn, dm, mf, dv, kw, dq, tc, id, tm, mh] |
| 15 | innovation*.mp. [mp=ti, ab, ot, nm, hw, fx, kf, ox, px, rx, ui, an, sy, tn, dm, mf, dv, kw, dq, tc, id, tm, mh] |
| 16 | invention*.mp. [mp=ti, ab, ot, nm, hw, fx, kf, ox, px, rx, ui, an, sy, tn, dm, mf, dv, kw, dq, tc, id, tm, mh] |
| 17 | improvement*.mp. [mp=ti, ab, ot, nm, hw, fx, kf, ox, px, rx, ui, an, sy, tn, dm, mf, dv, kw, dq, tc, id, tm, mh] |
| 18 | development*.mp. [mp=ti, ab, ot, nm, hw, fx, kf, ox, px, rx, ui, an, sy, tn, dm, mf, dv, kw, dq, tc, id, tm, mh] |
| 19 | method*.mp. [mp=ti, ab, ot, nm, hw, fx, kf, ox, px, rx, ui, an, sy, tn, dm, mf, dv, kw, dq, tc, id, tm, mh] |
| 20 | approach.mp. [mp=ti, ab, ot, nm, hw, fx, kf, ox, px, rx, ui, an, sy, tn, dm, mf, dv, kw, dq, tc, id, tm, mh] |
| 21 | principle*.mp. [mp=ti, ab, ot, nm, hw, fx, kf, ox, px, rx, ui, an, sy, tn, dm, mf, dv, kw, dq, tc, id, tm, mh] |
| 22 | 10 or 11 or 12 or 13 or 14 or 15 or 16 or 17 or 18 or 19 or 20 or 21 |
| 23 | safety.mp. [mp=ti, ab, ot, nm, hw, fx, kf, ox, px, rx, ui, an, sy, tn, dm, mf, dv, kw, dq, tc, id, tm, mh] |
| 24 | care.mp. [mp=ti, ab, ot, nm, hw, fx, kf, ox, px, rx, ui, an, sy, tn, dm, mf, dv, kw, dq, tc, id, tm, mh] |
| 25 | security.mp. [mp=ti, ab, ot, nm, hw, fx, kf, ox, px, rx, ui, an, sy, tn, dm, mf, dv, kw, dq, tc, id, tm, mh] |
| 26 | protection.mp. [mp=ti, ab, ot, nm, hw, fx, kf, ox, px, rx, ui, an, sy, tn, dm, mf, dv, kw, dq, tc, id, tm, mh] |
| 27 | 23 or 24 or 25 or 26 |
| 28 | incident*.mp. [mp=ti, ab, ot, nm, hw, fx, kf, ox, px, rx, ui, an, sy, tn, dm, mf, dv, kw, dq, tc, id, tm, mh] |
| 29 | error*.mp. [mp=ti, ab, ot, nm, hw, fx, kf, ox, px, rx, ui, an, sy, tn, dm, mf, dv, kw, dq, tc, id, tm, mh] |
| 30 | mistake*.mp. [mp=ti, ab, ot, nm, hw, fx, kf, ox, px, rx, ui, an, sy, tn, dm, mf, dv, kw, dq, tc, id, tm, mh] |
| 31 | fault*.mp. [mp=ti, ab, ot, nm, hw, fx, kf, ox, px, rx, ui, an, sy, tn, dm, mf, dv, kw, dq, tc, id, tm, mh] |
| 32 | blunder*.mp. [mp=ti, ab, ot, nm, hw, fx, kf, ox, px, rx, ui, an, sy, tn, dm, mf, dv, kw, dq, tc, id, tm, mh] |
| 33 | slip*.mp. [mp=ti, ab, ot, nm, hw, fx, kf, ox, px, rx, ui, an, sy, tn, dm, mf, dv, kw, dq, tc, id, tm, mh] |
| 34 | slipup*.mp. [mp=ti, ab, ot, nm, hw, fx, kf, ox, px, rx, ui, an, sy, tn, dm, mf, dv, kw, dq, tc, id, tm, mh] |
| 35 | complication*.mp. [mp=ti, ab, ot, nm, hw, fx, kf, ox, px, rx, ui, an, sy, tn, dm, mf, dv, kw, dq, tc, id, tm, mh] |
| 36 | failures*.mp. [mp=ti, ab, ot, nm, hw, fx, kf, ox, px, rx, ui, an, sy, tn, dm, mf, dv, kw, dq, tc, id, tm, mh] |
| 37 | event*.mp. [mp=ti, ab, ot, nm, hw, fx, kf, ox, px, rx, ui, an, sy, tn, dm, mf, dv, kw, dq, tc, id, tm, mh] |
| 38 | adverse event*.mp. [mp=ti, ab, ot, nm, hw, fx, kf, ox, px, rx, ui, an, sy, tn, dm, mf, dv, kw, dq, tc, id, tm, mh] |
| 39 | undesirable event*.mp. [mp=ti, ab, ot, nm, hw, fx, kf, ox, px, rx, ui, an, sy, tn, dm, mf, dv, kw, dq, tc, id, tm, mh] |
| 40 | hazard*.mp. [mp=ti, ab, ot, nm, hw, fx, kf, ox, px, rx, ui, an, sy, tn, dm, mf, dv, kw, dq, tc, id, tm, mh] |
| 41 | problem*.mp. [mp=ti, ab, ot, nm, hw, fx, kf, ox, px, rx, ui, an, sy, tn, dm, mf, dv, kw, dq, tc, id, tm, mh] |
| 42 | pitfall*.mp. [mp=ti, ab, ot, nm, hw, fx, kf, ox, px, rx, ui, an, sy, tn, dm, mf, dv, kw, dq, tc, id, tm, mh] |
| 43 | catastrophic.mp. [mp=ti, ab, ot, nm, hw, fx, kf, ox, px, rx, ui, an, sy, tn, dm, mf, dv, kw, dq, tc, id, tm, mh] |
| 44 | 28 or 29 or 30 or 31 or 32 or 33 or 34 or 35 or 36 or 37 or 38 or 39 or 40 or 41 or 42 or 43 |
| 45 | "high reliability organi*ation*".mp. [mp=ti, ab, ot, nm, hw, fx, kf, ox, px, rx, ui, an, sy, tn, dm, mf, dv, kw, dq, tc, id, tm, mh] |
| 46 | Resilient organi*ation*.mp. [mp=ti, ab, ot, nm, hw, fx, kf, ox, px, rx, ui, an, sy, tn, dm, mf, dv, kw, dq, tc, id, tm, mh] |
| 47 | Error free organi*ation*.mp. [mp=ti, ab, ot, nm, hw, fx, kf, ox, px, rx, ui, an, sy, tn, dm, mf, dv, kw, dq, tc, id, tm, mh] |
| 48 | High stable organi*ation*.mp. [mp=ti, ab, ot, nm, hw, fx, kf, ox, px, rx, ui, an, sy, tn, dm, mf, dv, kw, dq, tc, id, tm, mh] |
| 46 | organi*ation*.mp. [mp=ti, ab, ot, nm, hw, fx, kf, ox, px, rx, ui, an, sy, tn, dm, mf, dv, kw, dq, tc, id, tm, mh] |
| 48 | high reliability industrie*.mp. [mp=ti, ab, ot, nm, hw, fx, kf, ox, px, rx, ui, an, sy, tn, dm, mf, dv, kw, dq, tc, id, tm, mh] |
| 49 | high reliability bodies.mp. [mp=ti, ab, ot, nm, hw, fx, kf, ox, px, rx, ui, an, sy, tn, dm, mf, dv, kw, dq, tc, id, tm, mh] |
| 50 | high reliability association*.mp. [mp=ti, ab, ot, nm, hw, fx, kf, ox, px, rx, ui, an, sy, tn, dm, mf, dv, kw, dq, tc, id, tm, mh] |
| 51 | high reliability company.mp. [mp=ti, ab, ot, nm, hw, fx, kf, ox, px, rx, ui, an, sy, tn, dm, mf, dv, kw, dq, tc, id, tm, mh] |
| 52 | companies.mp. [mp=ti, ab, ot, nm, hw, fx, kf, ox, px, rx, ui, an, sy, tn, dm, mf, dv, kw, dq, tc, id, tm, mh] |
| 53 | aviation.mp. [mp=ti, ab, ot, nm, hw, fx, kf, ox, px, rx, ui, an, sy, tn, dm, mf, dv, kw, dq, tc, id, tm, mh] |
| 54 | "air industry".mp. [mp=ti, ab, ot, nm, hw, fx, kf, ox, px, rx, ui, an, sy, tn, dm, mf, dv, kw, dq, tc, id, tm, mh] |
| 55 | military.mp. [mp=ti, ab, ot, nm, hw, fx, kf, ox, px, rx, ui, an, sy, tn, dm, mf, dv, kw, dq, tc, id, tm, mh] |
| 56 | engineering.mp. [mp=ti, ab, ot, nm, hw, fx, kf, ox, px, rx, ui, an, sy, tn, dm, mf, dv, kw, dq, tc, id, tm, mh] |
| 57 | 45 and 46 and 47 and 48 and 49 and 50 and 51 and 52 and 53 and 54 and 55 and 56 |
| 57 | 9 and 22 and 27 and 44 and 57 |
